# Exosomes in inflammation and cancer: from bench to bedside applications

**DOI:** 10.1186/s43556-025-00280-9

**Published:** 2025-06-10

**Authors:** Shiyuan Huang, Fang Yan, Yi Qiu, Tao Liu, Wenjin Zhang, Yige Yang, Rao Zhong, Yang Yang, Xi Peng

**Affiliations:** 1 Sichuan Industrial Institute of Antibiotics, School of Pharmacy, Chengdu University, No. 2025, Chengluo Avenue, Chengdu, 610106 China; 2 Geriatric Diseases Institute of Chengdu, Department of Geriatrics, Center for Medicine Research and Translation, Department of Critical Care Medicine,, Chengdu Fifth People’s Hospital, The Second Clinical Medical College, Affiliated Fifth People’s Hospital of Chengdu University of Traditional Chinese Medicine,, Chengdu, China; 3 Chongqing Municipality Clinical Research Center for Endocrinology and Metabolic Diseases, Chongqing University Three Gorges Hospital, School of Medicine, Chongqing University, Chongqing, China; 4 Department of Critical Care Medicine, The First Affiliated Hospital of Chongqing Medical University, Chongqing, China; 5 Department of Gastroenterology, Sir Run Run Hospital, Nanjing Medical University, Nanjing, China

**Keywords:** Exosomes, Inflammation, Cancer, Microenvironment, Clinical translation

## Abstract

Exosomes, lipid bilayer nanovesicles secreted by nearly all cell types, play pivotal roles in intercellular communication by transferring proteins, nucleic acids, and lipids. This review comprehensively summarizes their multiple functions in inflammation and cancer. In inflammation, exosomes exhibit context-dependent pro- or anti-inflammatory effects: they promote acute responses by delivering cytokines and miRNAs to activate immune cells, yet suppress chronic inflammation via immunoregulatory molecules. Two representative inflammatory diseases, namely sepsis and inflammatory bowel disease, were highlighted to elucidate their roles in the acute and chronic inflammatory diseases. In cancer, exosomes orchestrate tumor microenvironment (TME) remodeling by facilitating angiogenesis, metastasis, and immune evasion through interactions with cancer-associated fibroblasts, tumor-associated macrophages, and extracellular matrix components. Furthermore, exosomes can facilitate the transition from inflammation to cancer by impacting pertinent signaling pathways via their transported oncogenic and inflammatory molecules. Tumor-derived exosomes also serve as non-invasive biomarkers correlating with disease progression. Clinically, exosomes demonstrate promise as therapeutic agents and drug carriers, evidenced by ongoing trials targeting inflammatory diseases and cancers. However, challenges in isolation standardization, scalable production, and understanding functional heterogeneity hinder clinical translation. Future research should prioritize elucidating cargo-specific mechanisms, optimizing engineering strategies, and advancing personalized exosome-based therapies. By bridging molecular insights with clinical applications, exosomes hold great potential in precision medicine for inflammation and oncology.

## Introduction

Inflammation represents a fundamental yet potent defensive response, swiftly acting to potential pathogens, mitigating further tissue damage, and stimulating repair mechanisms [1]. Inflammatory diseases, which can manifest in various systems and organs of the body, are generally categorized into two types: acute inflammation and chronic inflammation. Acute inflammation, typically induced by infection or injury, represents the most prevalent form of inflammation [2]. Presently, prevalent acute inflammatory conditions encompass sepsis, hepatitis, pancreatitis, myocarditis, and COVID-19 et al. [3–6]. Chronic inflammation is characterized by a persistent or recurrent inflammatory response that extends beyond the typical recovery period associated with acute inflammation [7]. Chronic inflammatory diseases, including inflammatory bowel disease (IBD), atherosclerosis, systemic lupus erythematosus, and non-alcoholic fatty liver disease, are major global causes of mortality globally [8]. Long-term chronic inflammation is associated with continuous activation of the immune system, which can result in cell damage, DNA mutations, and ongoing tissue repair. These processes may create a conducive environment for cancer development, thereby promoting its occurrence [1, 9].

Cancer is a disease characterized by uncontrolled cell proliferation and dysfunction due to genetic mutations, involving complex interactions between cancer cells and their microenvironment [10]. The tumor microenvironment (TME), comprising tumor cells, immune cells, fibroblasts, blood vessels, and extracellular matrix components, affecting cancer progression, metastasis, and treatment response [11]. Cancer can metastasize, spreading from the primary site to other areas via the blood or lymphatic system, forming metastatic tumors [12]. In 2020, there were over 19 million new cancer cases worldwide, resulting in almost 10 million deaths. Breast and lung cancers emerged as the most prevalent types, with prostate cancer being predominantly observed in men [13].

Exosomes are bio-nano spheres, approximately 40–100 nm in diameter, with lipid bilayer vesicles secreted by cells [14]. These were initially discovered in sheep reticulocytes in 1983 [15]. In 1986, Johnstone et al. formally designated these structures as “exosomes” to differentiate them from extracellular vesicles [16]. Exosome production is a capability shared by nearly all normal cell types, encompassing both mammalian and plant cells [17, 18]. Exosomes derived from mammalian sources can be categorized based on their cellular origin, primarily into stem cell-derived and immune cell-derived exosomes. It has been reported that tumor cells can also release exosomes [19, 20]. Exosomes have multiple biological roles in inflammatory diseases and cancer [21, 22]. In inflammation, exosomes can serve as crucial intercellular communication mediums that can either promote or inhibit the onset of inflammation [23, 24]. Moreover, exosomes can also influence the progression of inflammation by affecting its microenvironment. Exosomes can serve as mediators of intercellular communication, regulating key components in the tumor microenvironment, such as fibroblasts, the extracellular matrix, and tumor-associated macrophages [25]. Modifications in the tumor microenvironment, induced by exosomal regulation, can influence tumor progression and metastasis [26]. The above-mentioned research foundation provides a possibility of utilizing exosomes for diagnosis, therapy, and drug delivery in inflammation and cancer.

**Fig. 1:**
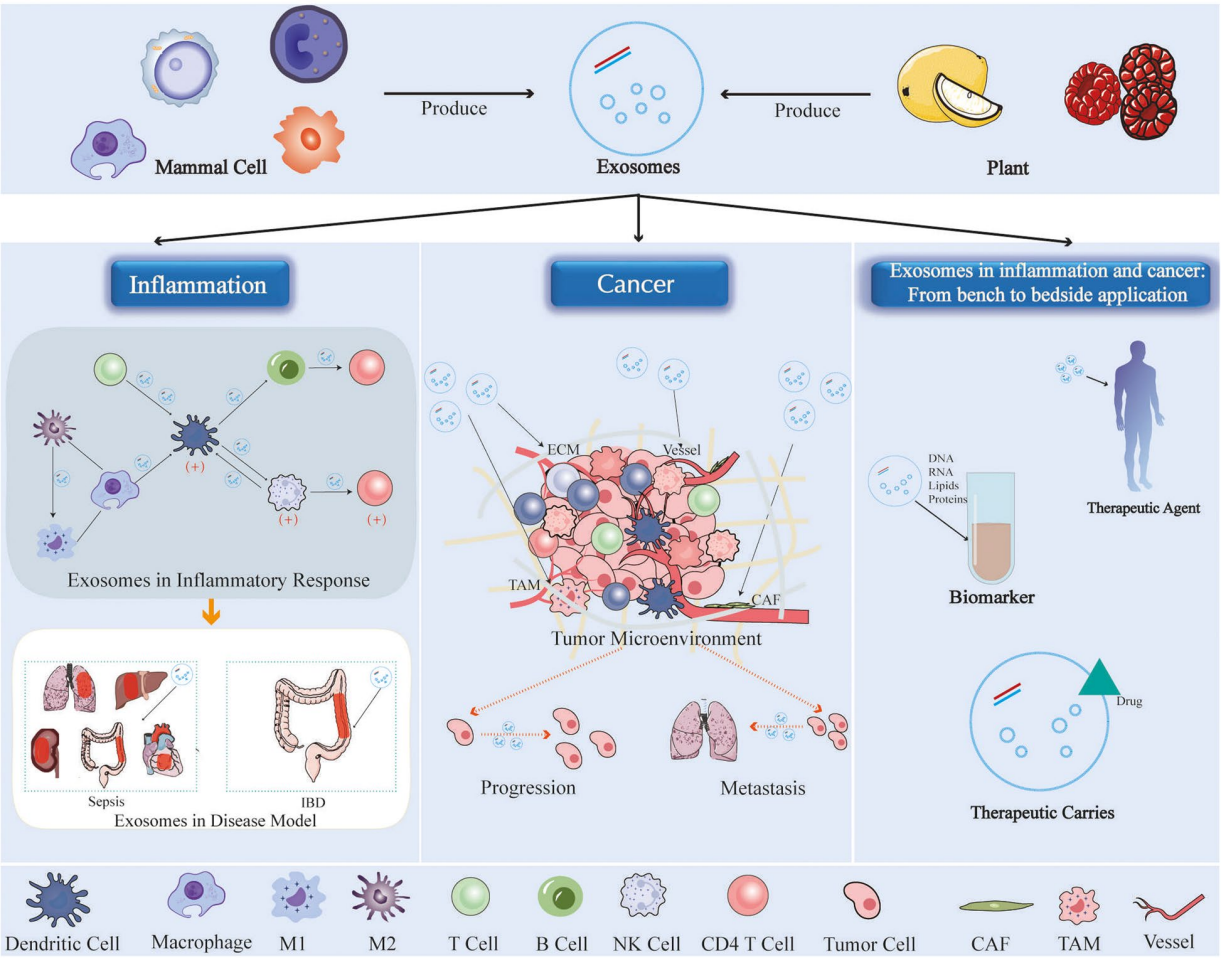
Exosomes in inflammation and cancer. Exosomes, which can be generated by various cells, exhibit a dichotomous role in inflammation, demonstrating both anti-inflammatory and pro-inflammatory effects. They possess the capacity to influence tumor progression via regulation of the tumor microenvironment. Furthermore, exosomes hold significant potential for application in diagnostics, therapeutic interventions, and drug delivery systems

The purpose of this review is to conduct a comprehensive analysis of the role of exosomes in inflammation and cancer, as well as their application from bench to bedside. We summarized the pro-inflammatory and anti-inflammatory effects of exosomes, the role of exosomes in sepsis (a representative of acute inflammation) and inflammatory bowel disease (a representative of chronic inflammation), and the function of exosomes in the inflammatory microenvironment. We concluded the functions of exosomes in the tumor microenvironment and in the transition from inflammation to cancer, and illustrated the potential of tumor-related exosomes as biomarkers. Additionally, based on data from www.clinicaltrials.gov and other relevant sources, we explored the current clinic-related application of exosomes as biomarkers diagnostics, therapeutic agent and drug carriers for inflammation and tumor. Although translation of exosomes from the bench to the bedside application necessitates surmounting numerous obstacles and challenges, it is believed that exosomes will usher in a new era of precise medicine in inflammation and cancer (Fig. 1).

## Biological function of exosomes

**Fig. 2:**
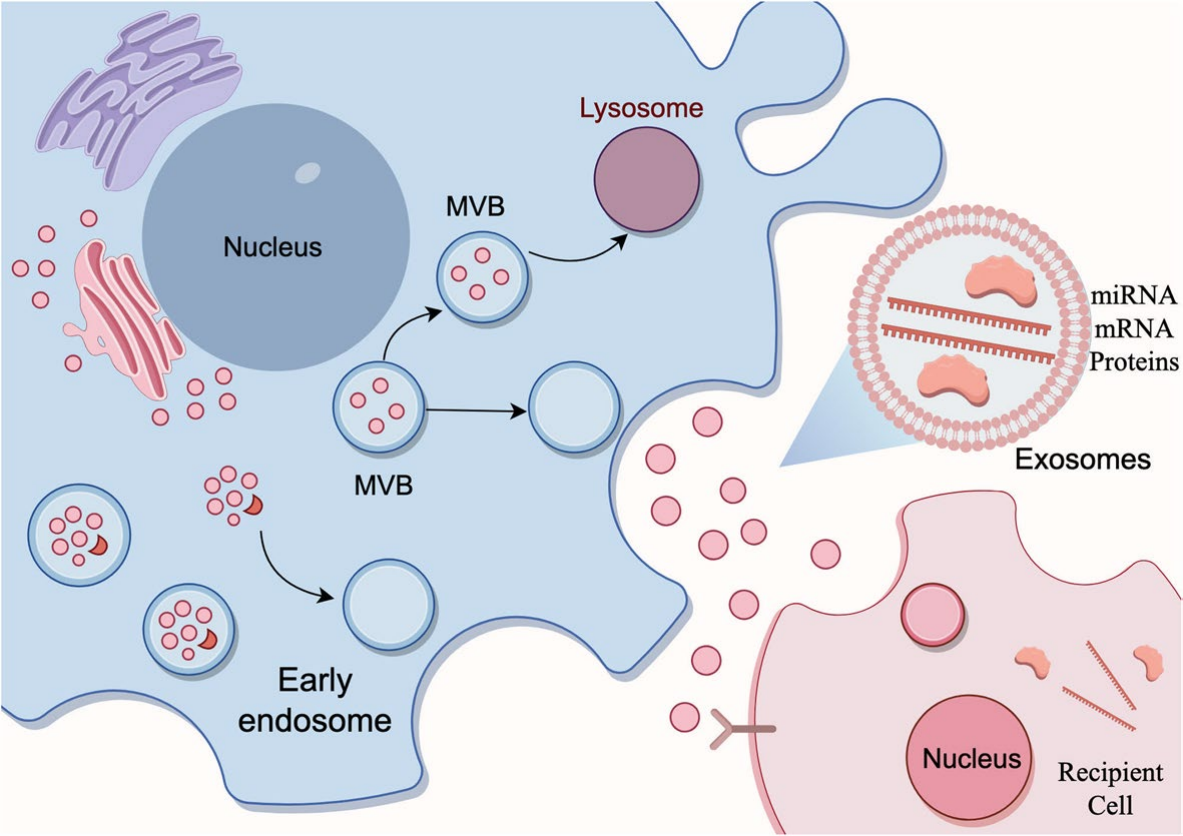
The biosynthesis of exosomes. The invagination of the cell membrane leads to the formation of early endosomes. These, upon maturation, give rise to late endosomes and subsequently, MVBs. Alternatively, late MVBs may either fuse with lysosomes for degradation or expel exosomes by fusing with the plasma membrane. The biogenesis of microvesicles takes place directly through budding from the plasma membrane, while apoptotic bodies are liberated during apoptosis. Exosomes contain a variety of miRNAs, mRNAs, and proteins. After being released, exosomes target and act on recipient cells

The formation of exosome goes through complex biological processes. Initially, the plasma membrane undergoes invagination to generate intracellular endosomes [27]. Subsequently, these endosomes further invaginate, resulting in the creation of multivesicular bodies (MVB) containing intraluminal vesicles (ILVs) ranging from 40 ~ 150 nm in diameter [28]. In the final stage, part of the MVB merges with the plasma membrane, releasing ILVs into the extracellular environment as exosomes [29] (Fig. 2).

Exosomes contain a variety of composition, primarily proteins, lipids, and RNA. The proteins include structure related proteins such as CD63, CD81 and HSC70, transporter protein such as Lysosome-associated membrane proteins (LAMPs), and function related proteins including some enzymes and some signal transduction protein (e.g. PI3 K, Ras) [30]. Among the lipids, cholesterol, phospholipids, and other varieties are presented [31]. Exosomes also harbor a multitude of RNAs, in which miRNA play crucial biological roles in regulating gene expression and facilitating intercellular communication [32]. This underscores their significance as a component within exosomes.

Exosomes, as intercellular communication mediums, regulate the physiological state of the target cells by recognizing surface receptors, fusing with the cells to release their contents, and finally exerting their intercellular functions [33]. Exosomes possess specific membrane proteins, such as tetraspanins (CD63, CD9, CD81) and integrins, which bind to receptors on target cells, aiding in exosome recognition. Upon internalization, exosomal cargo is delivered into target cells, influencing cellular signaling and gene expression in both health and disease through bioactive components like miRNA, mRNA, cytokines, and proteins [34, 35]. The miRNA within exosomes can bind to mRNA in target cells, either inhibiting its translation or accelerating its degradation [36]. Upon entering the recipient cell, the mRNA transported by exosomes can be translated into proteins to directly impact cell function. Regarding immune regulation, various cytokines, receptors, and antigen information carried by exosomes can interact with immune cells. Exosomes can interact with recipient cell receptors through their protein cargo, modulating specific signaling pathways by either activation or inhibition [37].

## Role of exosomes in inflammation

Exosomes serve a critical role in intercellular communication and exhibit a dual function in inflammation [38]. On one hand, they can promote the inflammatory response and accelerate the process of inflammation [22]. On the other hand, they can inhibit the inflammatory response, relieve the progression of inflammation, and mitigate the damage caused by inflammation [39]. In acute inflammation, chronic inflammation, and autoimmune diseases, exosomes consistently play a constructive role via modulating inflammatory microenvironment (IME) [22, 40, 41], which involve pro-inflammatory and anti-inflammatory progression [42].

### Promotion or suppression of inflammatory responses

#### Role of exosomes in promoting inflammation

Exosome-carried cytokines, chemokines, and miRNAs can activate and recruit immune cells, thereby enhancing local inflammation [43]. Exosomes play a crucial role in the loading and transport of cytokines, such as IL-1β, TNF-α, and IL-6. Under inflammatory stimulation, exosomes carrying IL-1β can activate macrophages, thereby exacerbating the inflammatory response [44, 45]. Furthermore, exosomes transport a range of chemokines, which can intensify the inflammation process by promoting the accumulation of immune cells [46]. For example, macrophage exosomes that contain CXCL2 can bind with neutrophils, facilitating the delivery of CXCL2. This interaction induces chemotaxis in the neutrophils, effectively recruiting them to the site of the inflamed tissue [47].

Exosomes transport miRNAs that induce inflammation by promoting the release of inflammatory factors and activating specific signaling pathways [48]. Serum exosomes containing miR-27-3p target PPARγ, activating microglial cells and elevating pro-inflammatory factors like IL-1β, IL-6, and TNF-α, thereby intensifying the inflammatory response [49]. Similarly, exosomes carrying miR-21 can also play a similar role. Exosomes carrying miR-21 can stimulate macrophages, induce their transformation to a pro-inflammatory phenotype, and down-regulate the target gene PDCD4, thereby reducing the release of inflammatory factors [50]. Recent research found that, mammary epithelial cells, upon infection with lipoteichoic acid of Staphylococcus aureus, produce exosomes containing high concentrations of miR-221. Exosomes are internalized by macrophages, where miR-221 facilitates M1 polarization by suppressing SOCS1 expression and activating STAT1 and STAT3 signaling pathways [51]. Wang et al. found that periodontal ligament stem cell-derived exosomes carry miR-143-3p, which targets PI3 Kγ in macrophages and promotes pro-inflammatory M1 macrophage polarization by inhibiting PI3 K/AKT signaling and activating NF-κB signaling, thereby exacerbating the inflammatory response [52].

Exosomes not only amplify inflammation by transporting their own inflammatory-related factors but also bolster immune responses through interaction with inflammatory cells [53]. Sarcoidosis exosomes can bind to monocytes and peripheral blood mononuclear cells (PBMCs), aiding in monocyte recruitment to inflammation sites. These exosomes subsequently trigger the release of pro-inflammatory cytokines, including IL-1β, IL-6, and TNF, from monocytes and PBMCs [54]. Exosomes from osteoarthritis patient chondrocytes can induce IL-1β secretion in LPS/ATP-treated PMA-induced THP1 macrophages [55]. Exosomes, released by macrophages, NK cells, and DCs, function as pro-inflammatory mediators via paracrine signaling. They influence target cells directly or in combination with cytokines and chemokines, creating synergistic effects on immune systems [56].

#### Role of exosomes in suppressing inflammation

Exosomes also transport anti-inflammatory agents, such as TGF-β and IL-10, which help mitigate inflammation by modulating the immune response [57]. Among the numerous of exosomes exhibiting anti-inflammatory effects, those originating from mesenchymal stem cells (MSCs) serve as the most representative [58]. Research indicates that MSCs-derived exosomes contain anti-inflammatory components like cytokines and immunoregulatory growth factors, such as TGF-β1, IL-6, IL-10, mRNA, and regulatory miRNAs [59]. MSCs-derived exosomes encapsulate miRNA-146a (miR-146a), an anti-inflammatory miRNA. Once inside macrophage cytosol, miR-146a inhibits the signaling pathway, reducing the production of inflammatory factors like TNF-α, IL-6, and IL-1β. Consequently, it inhibits the development of the macrophage M1 phenotype [60].

In certain instances of chronic inflammation, exosomes may suppress overzealous immune responses through the modulation of immune tolerance, activation of negative feedback mechanisms, and engagement of alternative pathways [61, 62]. MSCs-derived exosomes help suppress auto-reactive lymphocyte proliferation and enhance anti-inflammatory cytokine secretion. MSCs-derived exosomes exhibit immunosuppressive effects on T and B lymphocytes both in vitro and in vivo, while also enhancing Treg cell populations [63]. Consequently, exosomes inhibit overzealous immune responses. Furthermore, another study illustrated that exosomes transporting miR-192 reduce excessive in vivo inflammatory responses via a negative feedback mechanism. This process effectively diminishes the expression of IL-6 and CCL2 in macrophages stimulated by CL097 or R848 [64]. These studies establishe a fundamental basis for the utilization of exosomes in the treatment of autoimmune diseases. Exosomes inhibit chronic inflammation by participating in alternative pathways, of which the NF-κB pathway is the most representative. For instance, MSCs-derived exosomes can specifically target TLR4 with the carried miR-145-5p, inhibiting its expression and thereby reducing the TLR4-mediated inflammatory response. This indirectly inhibits the TLR4/NF-κB signaling pathway, reducing the production of pro-inflammatory factors like TNF-α, IL-1β, and IL-6 [65].

### Disease models

Recent research has revealed that exosomes play an active role in the development of various inflammatory diseases. They can both contribute to exacerbate and alleviate disease progression and damage [66]. In this section, we present two representative inflammatory diseases for elucidating the significance of exosomes within acute and chronic conditions.

#### Sepsis

Sepsis is a systemic inflammatory response syndrome induced by infection, which can lead to multiple organ dysfunction syndrome [67]. The current research on organ damage in sepsis mainly focuses on the lungs, kidneys, liver, and intestines [68].

Exosomes significantly contribute to the onset of sepsis. Studies have shown that secretory cells, such as macrophage, endothelial cell, intestinal epithelial cells, release exosomes containing various damage-associated molecular patterns (DAMPs), including High Mobility Group Box 1 (HMGB1), heat shock proteins, adenosine triphosphate (ATP), and extracellular RNA, among others [69]. DAMPs molecules can bind to pattern recognition receptors, especially Toll-like receptors, to trigger inflammatory signaling [70]. Moreover, during sepsis, certain exosomes can accelerate the disease’s progression due to the components they transport. In sepsis, macrophages secrete exosomes with elevated APN/CD13 expression, which can transmit inflammatory signals to lung epithelial cells. When APN/CD13 in these exosomes binds to the TLR4 cell surface receptor, it induces necrosis of lung epithelial cells, characterized by reactive oxygen species (ROS) production, mitochondrial dysfunction, and NF-κB activation. Furthermore, the absorption of APN/CD13 from exosomes by lung tissue initiates a pronounced inflammatory response, leading to lung tissue damage. This subsequently exacerbates the lung injury associated with sepsis [71]. Alveolar epithelial cell-derived exosomes can modulate alveolar macrophages by transferring miR-92a-3p, which activates the NF-κB signaling pathway and increases pro-inflammatory cytokine expression. This process consequently amplifies the lung injury induced by sepsis [72].

Exosomes not only exacerbate the progression of sepsis but also simultaneously release exosomes with anti-inflammatory properties, serving to mitigate the inflammatory response induced by sepsis [73]. These exosomes, which can exert anti-inflammatory and anti-damage effects, are mainly derived from MSC, Tregs, M2 macrophages, DCs, and contain anti-inflammatory factors such as IL-10, Arginase-1 (Arg-1) and anti-inflammatory miRNAs such as miR-21, miR-124 [74]. The utilization of exosomes in sepsis treatment has demonstrated promising results in the CLP model of sepsis in mice, the LPS cell model, and the organoid model [75–77]. Exosomes from MSCs containing miR-26a-5p can degrade metastasis-associated lung adenocarcinoma transcript 1 (MALAT1). This process aids in alleviating sepsis-related oxidative stress and liver injury. Furthermore, these exosomes can significantly decrease elevated liver enzyme levels, specifically reducing serum aspartate aminotransferase (AST) and alanine aminotransferase (ALT) associated with sepsis. This demonstrates their protective effect against liver injury induced by sepsis [78]. The miR-125b-5p in exosomes derived from MSCs can inhibit the expression of STAT3, thereby reducing the pyroptosis of macrophages. Exosomes can mitigate sepsis-induced lung inflammation by down-regulating inflammation-related proteins like IL-6, TNF-α, and IL-18 [79].

#### Inflammatory bowel disease

Inflammatory bowel disease is a chronic inflammatory condition of the intestine, provoked by an immune response from genetically predisposed hosts to pathogens. It is characterized by aberrant mucosal immune responses and a dysfunctional intestinal barrier [80]. Exosomes are pivotal in IBD for modulating immune cell activity and intestinal barrier function, thus controlling inflammation onset and progression [81].

During the acute phase of IBD development, exosomes are able to exert a pro-inflammatory effect by influencing intestinal immune cell functions and may further exacerbate damage by increasing local immune responses that lead to impaired intestinal barrier function. In the acute colitis model, serum exosomes can incite a pro-inflammatory reaction in macrophages. This is accomplished through the enhancement of p38 and ERK phosphorylation, alongside the generation of tumor necrosis factor [82]. Research has demonstrated that the let-7b-5p present in serum exosomes can activate macrophages. This leads to a significant rise in pro-inflammatory factor release and promotes macrophage proliferation. Furthermore, exosomes have been shown to enhance the permeability of Caco-2 cells in the intestine, while simultaneously reducing the integrity of the intestinal barrier. These effects collectively worsen the damage to the intestinal barrier observed in IBD [83]. During IBD, macrophages are not only influenced by other exosomes present in the body but also have the capability to secrete their own exosomes, which contain miRNA-223. It has been demonstrated that miR-223-loaded macrophage-derived exosomes have the capacity to inhibit TMIGD1 expression, thereby intensifying intestinal barrier dysfunction. Furthermore, these exosomes have been validated to worsen IBD symptoms in both mouse and human colon organoids when co-cultured with macrophages [84]

During the persistent phase of IBD, exosomes exhibit a more diversified role. On one hand, they can promote the maintenance of chronic inflammation by sustaining the activation state of immune cells [85]. On the other hand, they can also participate in regulating the intestinal microbiota, affecting its composition, thereby further influencing the immune response and inflammatory status of the intestine [86]. Zhao et al. discovered that in the IBD mouse model, exosomes containing elevated levels of dsDNA are present in the colon and plasma. T Macrophages internalize these exosomes, activating the STING pathway and increasing the expression of pro-inflammatory cytokines like TNF-α, IFN-β, and IL-6. This process further promotes the polarization of macrophages towards a pro-inflammatory M1 phenotype, exacerbating the inflammation [87]. Exosomes derived from the serum of TLR 2-deficient mice markedly diminish the activity of TLR 2/6 in probiotics (e.g., Bifidobacterium and Lactobacillus), which results in microbiota dysbiosis, subsequently exacerbating inflammation [88].

During the remission phase of IBD, exosomes exert a positive anti-inflammatory and reparative influence. These exosomes not only originate from within the body but also derive from external sources [89]. Notably, these exogenous exosomes have demonstrated beneficial effects in the treatment and repair of IBD [90]. Exosomes derived from human umbilical cord mesenchymal stem cells (hUC-MSCs), due to their TSG-6 protein content, can increase the expression of tight junction proteins and microvilli. These exosomes repair the damaged intestinal mucosal barrier in IBD and enhance the expression of anti-inflammatory cytokines like IL-4. Concurrently, they reduce the concentration of pro-inflammatory cytokines, thus modulating the immune response and mitigating intestinal inflammation. Furthermore, these hUC-MSCs exosomes can augment the proportion of Th2 and Treg cells in the intestinal mucosa, thereby contributing to the maintenance of immune equilibrium in patients with IBD [80]. Analogously, adipose-derived mesenchymal stem cells (Ad-MSCs) derived exosomes exhibit immunoregulatory properties in the context of acute colitis. Research indicates that AD-MSCs exosomes reduce pro-inflammatory cytokines (TNF-α, IFN-γ, IL-12, IL-17) and increase anti-inflammatory cytokines (TGF-β, IL-4, IL-10). Additionally, they have the capacity to modulate the immune response, shifting it from a Th1 type to a Th2 type, and reducing the quantity of Th17 cells, thereby mitigating the inflammatory response [91].

In addition, exosomes may further alleviate IBD pathology by affecting the diversity and abundance of microbiota [92]. Exosomal miR-181a, derived from MSCs, has been shown to alleviate symptoms of experimental colitis by bolstering intestinal barrier function. This is accomplished through its anti-inflammatory properties which also have the capacity to alter gut microbiota. It enriches gut microbiota diversity, prevents pathogen colonization, and promotes probiotic growth, enhancing overall gut health [93].

### Functions and impacts of exosomes in inflammatory microenvironments

**Fig. 3:**
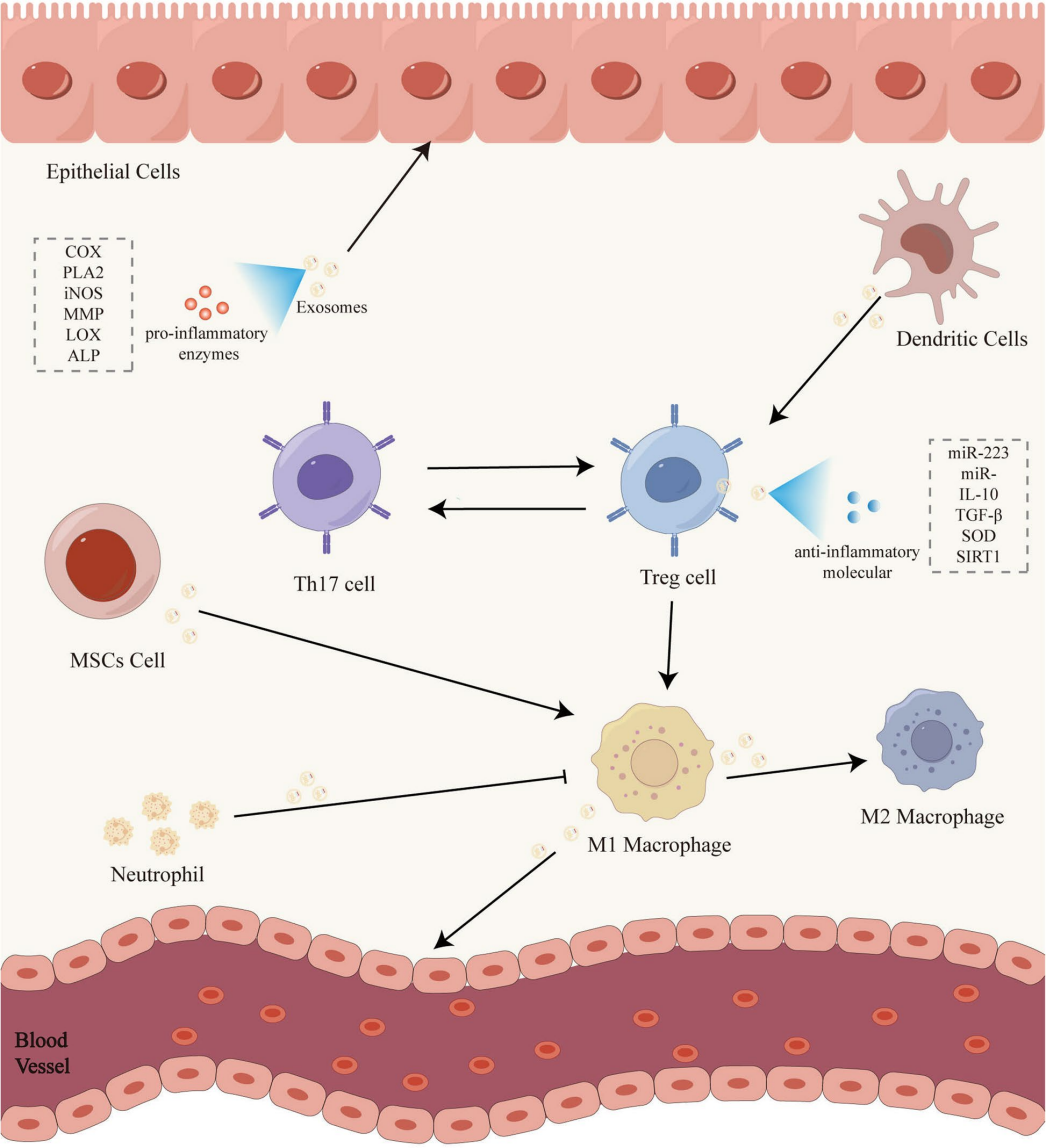
Exosomes in inflammatory microenvironments. The inflammatory microenvironment (IME) is collectively shaped by the elevated concentrations of pro-inflammatory enzymes, cells, and mediators present at the site of inflammation. Notably, exosomes predominantly impart either anti-inflammatory or pro-inflammatory effects on the IME by targeting specific inflammatory cells

Inflammatory microenvironments pertain to alterations in the microscopic milieu of local tissues or organs during an inflammatory response. Inflammation sites typically contain high levels of pro-inflammatory enzymes, cells, and mediators, collectively forming inflammatory microenvironments [94]. **(**Fig. 3**)** The primary method by which exosomes regulate the IME is by modulating the inflammatory cells within them. They can activate or inhibit specific inflammatory cells, affecting the balance and function of cell subpopulations [95]. In the IME, the main inflammatory cells include macrophages, dendritic cells, and lymphocytes, which play a significant role in shaping the inflammatory microenvironment [96]. Studies indicate that miR-30 d-5p in neutrophil-derived exosomes can promotes NF-κB signaling and M1 macrophage polarization by inhibiting SOCS-1 and SIRT1 in macrophages, thus advancing sepsis-related acute lung injury miR-30 d-5p [97]. Furthermore, certain exosome surfaces express T cell co-stimulatory molecules such as MCH I, MCH II, and CD81, which are instrumental in mediating antigen-specific interactions between B cells and T cells. Exosomes from dendritic cells display the T cell co-stimulatory molecule CD81 and enhance T cell activation by presenting T cell receptors on their surfaces [98].

Furthermore, exosomes possess the capacity to regulate the intercellular balance, thereby modulating the inflammatory microenvironment [99]. A notable example of this is the balance between Treg and Th17 cells. An imbalance in these cells significantly contributes to inflammation. A research demonstrated that exosomes from human umbilical cord mesenchymal stem cells (UCMSCs) facilitate the shift of T cells to an anti-inflammatory state by increasing TGF-β and IL-10 levels while decreasing IL-17 levels, thereby aiding in the restoration of the Treg/Teff balance. Exosomes possess tolerogenic molecules like PD-L1, TGF-β, and galectin-1, which inhibit T cell proliferation and enhance IL-10 and TGF-β production, promoting Treg cell generation. Exosomes indirectly modulate T cell responses via the adenosine A2 A receptor, while also inhibiting dendritic cell maturation and enhancing IL-10 production, which promotes Treg cell generation. This regulates the Th17/Treg balance, significantly impacting inflammation development [100].

Exosomes can also suppress the inflammatory reaction in the IME. Exosomes from immature dendritic cells can induce T cell anergy or deletion and activate CD4^+^ regulatory T cells. Treg cells mitigate the inflammatory microenvironment by releasing exosomes with anti-inflammatory molecules that curb IFN-γ secretion, inhibit CD4^+^ Th1 cell proliferation, and promote the differentiation of other T cells into Treg cells. CD4^+^ T cells and specific B cells with FasL-containing exosomes can trigger apoptosis in recipient T cells. ECs utilize exosomes to deliver anti-inflammatory miRNA, such as miR-146a, miR-223, miR-21, modulating T cell responses and preventing chronic inflammation [101].

In addition, exosomes can also influence the inflammatory microenvironment by regulating the expression and activity of pro-inflammatory enzymes. Enzymes like cyclooxygenase (COX), phospholipase A2 (PLA2), and inducible nitric oxide synthase (iNOS) play a role in regulating inflammatory responses [102–104]. Exosomes, serving as carriers for intercellular communication, transmit specific molecules, such as small RNAs, proteins, and lipids, between immune cells to affect the expression of pro-inflammatory enzymes within the inflammatory microenvironment. Wang et al. found that nasal irrigation fluid exosomes carrying mucin 5 AC can be taken up by fibroblasts, promoting a significant increase in COX-2 expression, which activates the COX-2/PGE2 pathway to promote MMP-9 and vascular endothelial growth factor (VEGF) exacerbating sinusitis [105]. Bone marrow mesenchymal stromal cells (BMMSCs) -derived exosomes can suppress COX-2 expression, thereby inhibiting autophagy mediated by the PI3 K/AKT/mTOR signaling pathway. Subsequent to the suppression of COX-2 expression, exosomes further mitigate the IL-1β-induced inflammatory response [106].

## Role of exosomes in cancer

### Exosome-related tumor microenvironment

**Fig. 4:**
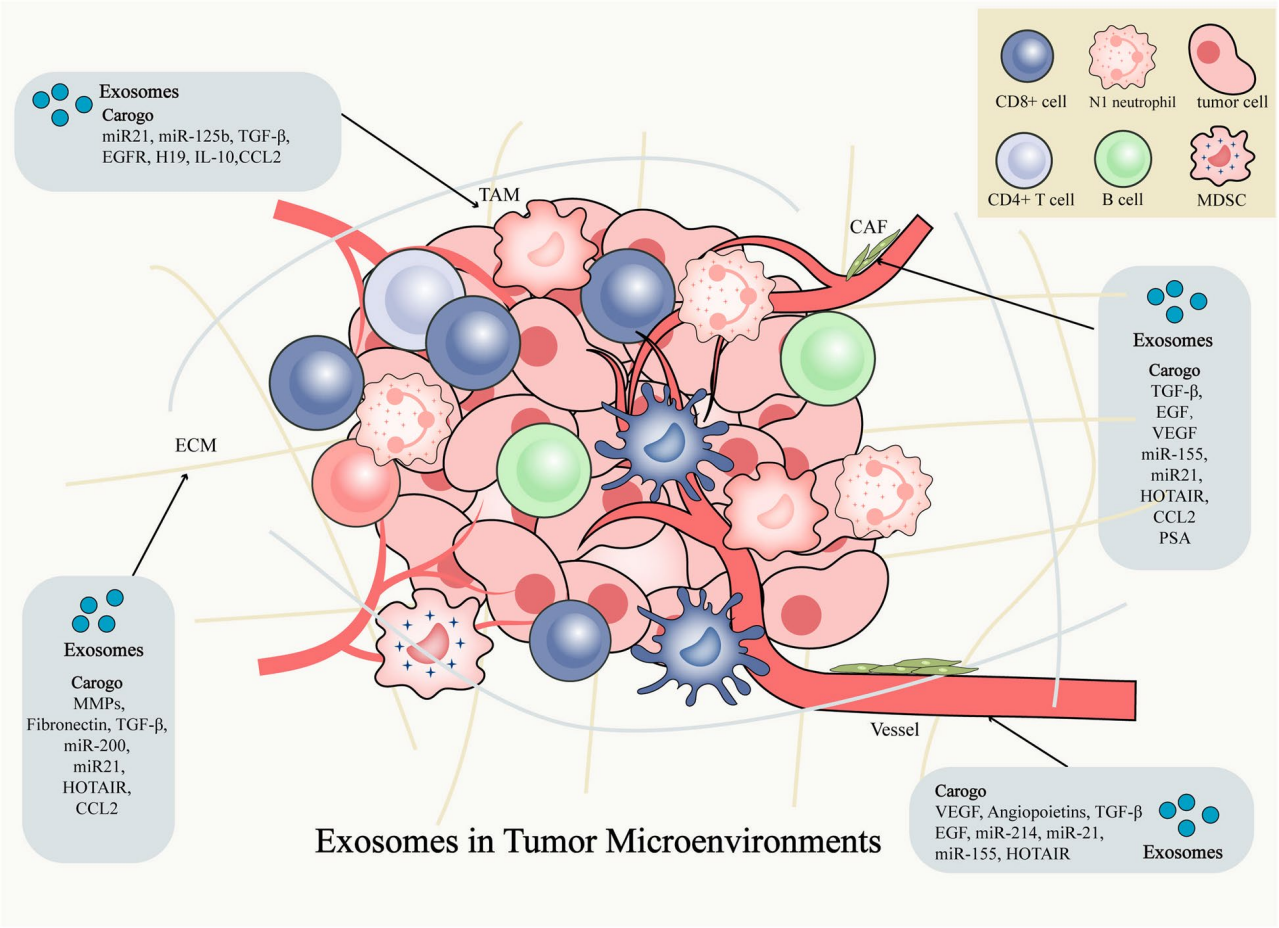
Exosomes in tumor microenvironments. Exosomes exert signaling functions by influencing key cellular components in the TME, such as the ECM, CAF, TAM, and blood vessels, through their carried cargo. ECM, extra cellular matrix; CAF, cancer-associated fibroblasts; TAM, tumor-associated macrophage

Tumor migration and progression are influenced not only by the tumor cells but also by the TME, comprising various non-malignant cells and non-cellular components [107]. The former, includes fibroblasts, endothelial cells, pericytes, and immune cells, while the latter contains the extracellular matrix, soluble factors, and exosomes [108, 109]. Exosomes play multifaceted functions in TME depending on their carried proteins, lipids and RNA [110–113] (Fig. 4).

Exosomal proteins mainly include enzymes, receptors, signaling molecules, and immunoregulatory proteins that function by directly activating target cell pathways or modulating the components of the TME. Exosomal proteins affect tumor invasion and metastasis, immune evasion, and angiogenesis by affecting the TME. Exosomes contain extracellular matrix (ECM) degrading enzymes, such as matrix metalloproteinases (MMPs), cathepsins [114], hyaluronidase [115], collagens, laminins, and fibronectin [116], which promote the invasive and metastatic ability of cancer cells by directly degrade the ECM and basement membrane or/and promoting epithelial-mesenchymal transformation (EMT) [117]. In addition, the exosomal membrane can carry integrins, which interact with ECM and participate in the regulation of cell adhesion process to promote tumor invasion and metastasis.

Exosomes also have enriched immune checkpoint proteins (such as PD-L1 [118], CD47 [119], LAG-3 [120]), which can bind with the receptors on the surface of immune cells, mediating immune escape. In addition, exosomes can also carry angiogenesis-enhancing factors, such as VEGF [121] and bFGF [122], to regulate TME by affecting the vascular system which mainly promotes the supply of oxygen and nutrients necessary for tumor proliferation and metastasis [122, 123]. Exosomes carrying VEGF may be crucial for early tumor angiogenesis [122, 124]. Glioblastoma (GBM)-derived exosomes carrying VEGF-C can directly promote angiogenesis by binding to VEGFR2 on vascular endothelial cells. And GBM exosomes also inhibit the Hippo signaling pathway by releasing VEGF-C, thereby increasing the expression of TAZ, a key vascular regulator, ultimately promoting angiogenesis [125].

The lipid components of exosomes mainly maintain the stability of the exosome membrane, protect the contents from damage during transport, and target them for delivery to the target cells. Furthermore, exosomes possess the ability to transport lipid signaling molecules, thereby directly influencing the tumor microenvironment. Fatty acids (FAs) found in glioma-derived exosomes stimulate a metabolic shift towards oxidative phosphorylation. This process results in DC immunosuppression and the initiation of ferroptosis in mature DCs (mDCs) via the NRF2/GPX4 axis. Consequently, it fosters the proliferation of glioma [126].

Exosomal functional RNA molecules, including mRNA, miRNA, and lncRNA, significantly contribute to the regulation of the tumor microenvironment. Exosomes can partially retain the genetic information of the parent cell, therefore, some tumor-derived exosomes can transfer oncogenes to recipient cells and transform them towards a tumor phenotype. For example, breast cancer exosomes carrying miR-21 inhibit PTEN expression, activate the PI3 K/AKT pathway in recipient fibroblasts, prompting their transformation into cancer-promoting tumor-associated fibroblasts (CAFs), which secrete IL-6 and MMP2, accelerating matrix degradation [127]. Exosomal RNAs also can enhance tumor invasion by inducing EMT in the TME. For instance, miR-335-5p carried by metastatic CRC cell lines derived exosomes can activate EMT and RAS signaling through its target gene RASA1 to promote CRC cell invasion and metastasis [128]. And cancer-associated fibroblast-derived exosomal lncRNA LINC00659 can promote the progression of EMT in CRC cells via the miR-342-3p/ANXA2 axis [129]. In addition, the RNAs in exosomes could also induce immune escape in the TME by regulating immune cell functions and inhibiting anti-tumor immune responses. LncRNA KCNQ1OT1 in colorectal cancer cell-derived exosomes can regulate the ubiquitination of PD-L1 through the miR-30a-5p/USP22 pathway, inhibiting the response of CD8^+^ T cells, allowing CRC cells to evade immune surveillance, thereby promoting the development of CRC [130]. MiR-21-5p in hepatocellular carcinoma cell (HCC)-derived exosomes promotes the polarization of TAM towards M2 phenotype by regulating the SP1/XBP1 signaling pathway, which leads to immunosuppressive state in the TME, enabling tumor cells to escape the attack of the immune system, and thus promoting the development and deterioration of HCC [131]. The exosomal lncRNA lncARSR, present in exosomes derived from renal cell carcinoma (RCC), can interact with miR-34/miR-449. This interaction augments the expression of STAT3 in RCC cells and mediates macrophage polarization, thereby facilitating tumor growth [132].

Exosomes from tumor or stromal cells can promote drug resistance by transferring miRNAs [133]. Exosomal miR-425-3p in cisplatin-resistant non-small cell lung cancer (NSCLC) promotes autophagy activation by targeting the AKT1/mTOR pathway, leading to cisplatin resistance in sensitive cells [134].

### Role of exosomes in cancer progression and metastasis

Exosomes can interact with critical components in the TME, including the ECM, cancer-associated fibroblasts (CAF), immune cells such as tumor-associated macrophages (TAM), and blood vessels to play a multifaceted role in tumor progression and metastasis [135]. They can not only manipulate the TME to aid in cancer growth and dissemination, but also regulate anti-tumor response [136].

Exosomes facilitate interactions between tumor cells and between tumor and normal cells by enabling the transfer of oncogenes and oncogenic signals from one tumor cell to another, or from tumor cells to normal cells [137]. For example, the transmission of EGFR-mutated exosomes to alveolar cells can lead to their transformation into lung cancer cells [138].

Exosomes regulate the gene expression of CAFs via the components they transport, thereby fostering their progression towards a tumor-promoting phenotype. For example, exosomal miRNA-146a derived from breast cancer cells induces the activation of CAFs by downregulating TXNIP to active Wnt/β-catenin axis, which promotes breast cancer progression [139]. Similarly, lung cancer cell-derived exosome ITGB6 can be taken up by fibroblasts and induce the activation of KLF10 positive feedback loop and TGF-β pathway, thereby inducing the activation of CAFs [140]. Activated CAFs are able to secrete exosomes carrying MMPs, which can directly degrade collagen and other matrix components in the ECM, breaking down the matrix barrier to promote cancer cell invasion and metastasis. ECM, a critical component of the tumor microenvironment, serves dual functions showing support or inhibit the progression of tumors [141]. Exosomes can influence the progression of cancer by mediating intercellular communication and regulating the remodeling of the ECM [117]. ECM provides structural support for tumor cells, affecting the ECM can influence the progression and migration of tumors [142]. For example, in gastric cancer, CAF-derived exosomes carry MMP11, enhancing the migration ability of tumor cells [143]. Some studies have found that the ECM stiffening stimulates the production of exosomes carrying more Jagged1, the key ligand for Notch activation, which can activate Notch signaling in recipient cells. The Notch pathway can promote the growth of tumors, including HCC [144]. Furthermore, tumor cell-derived exosomes possess the ability to degrade collagen fibers through the release of stromal effector molecules which can modify the density and architecture of the ECM, subsequently influencing the invasive potential of cancer cells [145].

Numerous studies have confirmed that exosomes from CAF enhance the mesenchymal characteristics of epithelial cells [146, 147]. This process is facilitated through the transfer of pro-EMT compounds such as TGF-β, thereby contributing to the induction and metastasis of EMT [148]. CAFs have the potential to release SNAI1-containing exosomes, which are then delivered to recipient lung cancer cells, thereby promoting EMT. Exosomal SNAI1 is essential for triggering EMT in lung cancer cells [149].

Tumor cell invasion and migration in vivo fundamentally depend on the execution of EMT [150]. EMT is a reversible process that transforms epithelial cells into a quasi-mesenchymal state, characterized by the loss of adhesion, apical-basal polarity, and basal anchoring, alongside the acquisition of migratory and invasive abilities [151]. In tumor cells, EMT triggers aggressive and metastatic characteristics, culminating in malignancy. Exosomes, enriched with small noncoding RNAs or miRNAs—now widely acknowledged as potent gene expression modifiers—enable cells to rapidly adapt to new environments, thereby directly or indirectly influencing EMT [152]. Exosomes carrying related proteins can also indirectly influence the process of EMT. For example, exosomes carrying MMP-1 can directly degrade the ECM and basement membrane, and can also bind to PAR1 protein, activating G protein signaling pathways, promoting EMT, thereby promoting breast cancer cell invasion and migration [153].

In the process of tumor development and metastasis, immune escape plays a crucial role [154]. “Immune escape” describes how tumor cells avoid detection and attack by immune cells, allowing them to survive and form metastatic tumors. Immune escape occurs when tumor cells express ligands and release immunosuppressive factors, inhibiting immune cell functions and depleting their population. The immunosuppressive microenvironment formed by immune escape plays a vitally important role in cancer progression and even in the response to immunotherapy [155]. Exosomes can transmit relevant signals to immune cells, hindering their antitumor functions by suppressing immune effector cell activity. This process promotes tumor progression and facilitates tumor escape. These exosomes carry immunosuppressive molecules that affect the development, maturation, and antitumor activity of immune cells, either directly or indirectly [156]. Exosomes derived from metastatic melanoma express the apoptosis-inducing molecule PD-L1 on their surface. Numerous studies have confirmed that exposure to IFN-γ results in an upregulation of PD-L1 exosomes. This process suppresses CD8 ^+^ T cell activity, facilitating tumor growth. Studies have demonstrated that IFN-γ exposure increases PD-L1 exosome levels, inhibiting CD8^+^T cell function and promoting tumor growth. Cancer exosomes carry tumor antigens that can trigger apoptosis in antigen-specific CD8^+^T cells, thereby suppressing tumor immunity [157]. Exosomes containing LGALS9 in the cerebrospinal fluid of glioblastoma patients can inhibit antigen presentation by dendritic cells and suppress T cell immunity [158]. Kidney cancer-derived exosomes enhance myeloid-derived suppressor cells’ ability to process and present tumor-associated antigens, thus activating T lymphocyte immune responses. Additionally, the signals contained within these exosomes can counteract the impairment of the JAK/STAT pathway induced by tumor-derived exosomes. This consequently enhances the cytotoxicity of T lymphocytes towards tumor-specific responses, fostering tumor growth and enabling evasion of immune surveillance [159]. In addition to inducing the differentiation of fibroblasts into myofibroblasts and regulating the expression of ECM components, TGF-β1 exhibits suppressive effects on the immune cell response, particularly that of NK cells [160]. Exosomes from hypoxic cancer cells deliver TGF-β1 to NK cells, reducing NKG2D receptor expression and impairing NK cell function and antitumor activity. Moreover, miR-23a present in these hypoxic tumor-derived exosomes directly suppresses CD107a expression in NK cells, facilitating immune evasion [161].

In addition, exosomes can also exert regulatory effects on immunity in the TME by affecting the polarization of TAMs, influencing tumor progression and metastasis [162]. For instance, exosomal miR-934 and miR-222-3p can induce the polarization of TAMs toward the M2 phenotype for anti-tumor, while miR-125b-5p can promote the M1 phenotype polarization for helping tumor. Moreover, exosomes also modulate the function of TAMs by transferring signaling molecules such as PD-L1 and IL-6, thereby affecting the immune response. These exosomal-mediated effects contribute to establishing an immunosuppressive environment conducive to tumor progression. Moreover, exosomes can transmit immunosuppressive signals, assisting CAFs in suppressing immune responses in the TME, and facilitating tumor cells in evading attacks from the immune system. Under specific conditions, such as hypoxia, the discharged miRNA and metabolic products can augment the stem cell attributes of CAFs, impede cell apoptosis, and stimulate cell survival as well as the formation of tumor spheroids [163].

During tumor migration, exosomes may also exert influence by contributing to angiogenesis [164]. Tumors generate new blood vessels to facilitate the supply of oxygen and nutrients, subsequently promoting tumor growth and progression [165]. VEGF is a key regulator that induces vascular permeability and initiates angiogenesis [166]. Exosomes can promote angiogenesis by transferring VEGF directly or by modulating the VEGF signaling pathway. In addition, exosomes can also carry factors or miRNAs other than VEGF to promote the formation of tumor blood vessels. Exosomes can carry various miRNAs, including miRNA-181a, miRNA-1246, miRNA-296, miRNA-494, and miRNA-155, to promote angiogenesis in cancer [167]. For example, exosomal miR-181-5p stimulates fibroblast activation via the upregulation of MMP-9 and facilitation of Smad2/3 phosphorylation, thereby promoting angiogenesis in colorectal cancer [168]. TGF-β, particularly in exosome-rich forms, significantly influences angiogenesis. Tumor-derived exosomes containing TGFβ accumulate in the TME, where they promote angiogenesis and drive head and neck squamous cell carcinoma (HNSCC) progression through TGF-β signaling [169]. Hypoxia significantly influences exosome formation, release, composition, and cancer angiogenesis [170]. Tumor cells under hypoxic conditions secrete a substantial number of exosomes that enhance angiogenesis by increasing protease-activated receptor 2 (PAR2) expression in epithelial cells. Hypoxia induces lung cancer cells to produce more exosomes enriched with miR-23a. As a result, it inhibits prolyl hydroxylase domain-containing proteins 1 and 2 (PHD1 and PHD2), causing hypoxia-inducible factor 1-α (HIF1 A) to accumulate in endothelial cells [171]. In addition, exosomes containing integrins can also assist tumor cells in metastasis and colonization of other organs [172]. TDE-derived integrins α5β1, α2 and αvβ3 can modify the local microenvironment to promote tumor metastasis to the liver [173].

### The key role of exosomes in the transition from inflammation to cancer

Exosomes, as key mediators of intercellular communication, play the role of messengers in the interaction between inflammation and cancer [174, 175]. Emerging evidence highlights their key role in the transition from inflammation to cancer. In chronic inflammation, the slow release of ROS can lead to genetic mutations in nearby cells, promote the proliferation of malignant transformed cells, and inhibit cell apoptosis. Exosomes can promote tumor progression by carrying pro-tumoral factors and miRNAs, inducing ROS expression, and activating related signaling pathways [176]. For instance, exosomes derived from pancreatic cancer can increase ROS levels through the pro-oxidative factors they carry and activate the STAT3 pathway to promote monocyte survival and arginase expression, exacerbating oxidative stress, inhibiting T cell function, and disrupting antigen presentation capabilities, thereby facilitating the immune evasion and tumor advancement of pancreatic cancer [177]. Furthermore, exosomes can induce DNA damage and genetic instability in chronic inflammation by transferring specific biomolecules (such as miRNA, circRNA, DNA, etc.), thereby promoting the malignant transformation of cells [66]. For example, arsenate induces hepatocyte-secreted exosomal circRNA_100284 which inhibits EZH2 expression by binding to miRNA-217 and in turn accelerates cell cycle progression (such as increase of S phase proportion), leading to increased DNA replication stress and accumulation of genetic errors. This mechanism plays a key role in the malignant transformation of hepatocytes caused by chronic arsenic exposure [178].

Simultaneously, tumors can release exosomes containing DAMPs such as HMGB1 and S100 A8/A9, continuously activating the TLR/NLRP3 inflammasome of immune cells, forming “inflammation-tumor” positive feedback loop [179, 180]. This regulatory relationship indicates that exosomes not only promote tumor progression by triggering chronic inflammation, but also facilitate tumor metastasis through their mediation of pre-metastatic microenvironment formation. [175, 181].

Numerous studies are currently exploring the role of exosomes in the transition from inflammation to cancer. The majority of these investigations concentrate on exosome-mediated inflammation signaling or carcinogenesis, fostering the creation of an immunosuppressive microenvironment and establishing a pre-metastatic tumor microenvironment [66, 175]. Paying attention to the role of exosomes in the transition from inflammation to cancer can better explore their potential as early markers of the transition from inflammation to cancer and can also provide new insights for targeted cancer treatments. Future research should deepen our understanding of the mechanisms by which exosomes facilitate this transition, while also advancing their clinical application.

### Potential of tumor-associated exosomes as biomarkers

Exosomes derived from tumors are prevalently found in the bloodstream and other bodily fluids, including urine [182]. Exosomes can effectively encapsulate molecular and genetic materials such as proteins, lipids, and nucleic acids, which mirror the composition of the originating tumor cells and avoid degradation during transportation [183]. Consequently, tumor-derived exosomes hold significant potential as biomarkers because of their widespread distribution and possibilities of non-invasive liquid biopsies [184].

Comprehensive studies have demonstrated that distinct differences in exosomal cargo are strongly correlated with the progression of cancer [137]. The concentrations of tumor derived exosomal proteins in cancer patients surpass those of healthy donors and tend to rise in patients with advancement of the disease. Notably, some membrane proteins, such as tetraspanin proteins including CD9, CD63, and CD81 et al., can serve as strong prognostic biomarkers for various cancers [185]. Gu et al. used ExoCounter system to analyze the serum of CRC patients, revealing elevated levels of exosomal CD147/CD9 compared to healthy individuals [186]. A Tim4-based sandwich enzyme-linked immunosorbent assay was utilized to measure serum levels of CD63 +, CD41 +, and CD61^+^ exosomes in individuals with pancreatic ductal adenocarcinoma (PDAC) and healthy controls, revealing significantly elevated levels in PDAC patients. Moreover, there was a significant difference in CD63^+^, CD41^+^, and CD61^+^ exosomes between early-stage PDAC and advanced-stage PDAC. Post-tumor resection, serum levels of CD63^+^, CD41^+^, and CD61^+^ exosomes decreased, suggesting a correlation between exosome count in serum and tumor burden [187].

Furthermore, the miRNA present in exosomes displays pronounced dysregulation across various malignant tumors. These exosomes effectively mirror the expression patterns of miRNA found in their originating tumor cells [188]. Research indicates that elevated miR-21 levels in circulating exosomes could be a biomarker for several malignant tumors, such as liver, stomach, breast, colorectal, ovarian, and esophageal cancers. Furthermore, elevated exosomal miR-21 levels in urine have been linked to bladder and prostate cancers [189]. Xiao et al. discovered that seven miRNAs (miR-23a, miR-1246, let-7a, miR-1229, miR-150, miR-223, and miR-21) are predominantly expressed in the serum exosomes of patients with Colorectal Cancer (CRC). Notably, the concentrations of these miRNAs diminish significantly following tumor resection. This observation suggests that these exosomal miRNAs could serve as promising biomarkers for CRC [190].

## From bench to bedside application

### Exosomes as biomarker

As previously noted, exosomes are present in various bio-fluids and carry nucleic acids, proteins, metabolites, and lipids from their parent cells. These characteristics allow them to serve as a reflection of the cellular state under both normal and pathological conditions [191]. In biopsies—including fluids, plasma, serum, urine, and saliva—exosomes have been employed to ascertain the patient’s diagnosis, prognosis, progress, and chemotherapy resistance status. Notably, an upregulated secretion of exosomes has been observed in numerous complex diseases, such as inflammation and cancer. During pathological states, cellular modifications can be tracked through the exosomes they release. The variances in the concentrations of specific molecules can be discerned and evaluated using transcriptomics, proteomics, and lipidomics research [192]. The potential use of exosomes in disease diagnosis has been particularly explored for the early detection and prognosis of inflammation and cancer (Table 1).

**Table 1:** Exosome biomarkers used for diagnosis and treatment monitoring in inflammation and cancer

Biomarker in Exosomes	Disease	Origin	Bio sample	Ref
CD63, CD9	Sepsis	Plasma exosomes	Plasma	[193]
BRD4, METTL3-eIF3 h, TOXW	Sepsis	Plasma exosomes urinary exosome	Plasma	[194]
HMGB1, SPTLC3, ATF3, HSPs, CXCL9, CXCL10, MPO, PRDX3, SOD2, FOXM1, SELS, GLRX2	Sepsis	Circulating exosomes	Blood	[195]
HIF-1α	Bacterial peritonitis	Serum exosomes	Serum	[196]
lncRNAs: TCONS_I2_00013502 and ENST00000363624	Rheumatoid arthritis	Serum exosomes	Serum	[197]
miR-885-5p, miR-6894-3p, miR-1268a	Rheumatoid arthritis	Serum exosomes	Serum	[198]
hsa_circ_0003914	Rheumatoid arthritis	Serum exosomes	Serum	[199]
miRNA (let-7a-5p, let-7b-5p, let-7 d-5p, let-7f-5p, let-7 g-5p, let-7i-5p, miR-128-3p, miR-25-3p)	Rheumatoid arthritis	Plasma exosomes	Plasma	[200]
miR-21, miR-146a and miR-155	Systemic lupus erythematosus	Circulating exosomes	Blood	[201]
tRF3-Ile-AAT-1, tiRNA5-Lys-CTT-1	Lupus nephritis	Urinary exosomes	Urine	[202]
hsa-miR-4796-5p, hsa-miR-7974	Lupus nephritis	Circulating exosomes	Blood	[203]
miR-146a	Lupus nephritis	Urinary exosomes	Urine	[204]
lncRNA PVT1, LINC00960, hsa-miR-107	Type 1 diabetes	Human pancreatic islet exosome	Blood, urine	[205]
lncRNAsMALAT1, LNC-EPHA6, LNC-RPS24, LIPCAR	Type 1 diabetes	Circulating exosomes	Blood, urine	[206]
PPI mRNA	Type 1 diabetes	β-Cells exosomes	Islet supernatant	[207]
miR-145-5p, miR-27a-3p	Diabetic kidney disease	Urinary exosomes	Urine	[208]
hsa-miR-146a-5p, hsa-miR-223-3p, hsa-miR-21-5p	Latent autoimmune diabetes	Plasma exosomes	Plasma	[209]
miRNAslet-7b-5p, miR-1290, miR-34a-5p, miR-3648	Sjogren syndrome	Salivary exosomes	Salivary	[210]
miRNA-127-3p, miRNA-409-3p, miRNA-410-3p, miRNA-541-5p, miRNA-540-5p	Sjogren syndrome	Serum exosomes	Serum	[211]
ITGAM, OLFM4, RAB10, CD36	Sjogren syndrome	Serum exosomes	Serum	[212]
circ-IQGAP2, circ-ZC3H6	Sjogren syndrome	MSC exosomes	MSG, blood	[213]
tRNA-Ile-AAT-2–1	Sjogren syndrome	Salivary exosomes	Salivary	[214]
miRNAs hsa-miR-203a-3p, hsa-miR-181a-5p, hsa-miR-181b-5p	Sjogren syndrome	Tear exosomes	Tear	[215]
Let-7 g-5p, miR-18a-5p, miR-145-5p, miR-374a-5p, miR-150-5p, miR-342-3p, miR-15a-5p, miR-19b-3p, miR-432-5p, miR-17-5p	Multiple sclerosis	Cerebrospinal fluid and serum exosomes	Cerebrospinal fluid and blood	[216]
CD31, CD105, CD144, MAL	Multiple sclerosis	CNS exosomes	Blood	[217]
miR-320a, miR-25-3p, miRNA, miR-191-5p, miR-145	Multiple sclerosis	Cerebrospinal fluid and serum exosomes	Blood and cerebrospinal fluid	[218]
LC–MS/MS	Inflammatory bowel disease (IBD)	Serum exosomes	Serum	[219]
miR-101, miR-21, miR-31, miR-142-3p, miR-142-5p, PSMA7	IBD	Salivary exosomes	Salivary	[220]
IL-6, TNF-α	IBD	Salivary exosomes	Salivary	[221]
NHP2, OLFM4, TOP1, SAMP, TAGL, TRIM28	Colorectal cancer	Tumor exosomes	Colon cancer tissue	[222]
CD9, CD63, CD81 and TSG101	Cancer	Tumor exosomes	Plasma	[223]
KRAS-G12D	Pancreatic cancer	Tumor exosomes	Blood	[224]
miR-21, miR-10b/miR-92a-3p	Colorectal cancer	Tumor exosomes	Plasma	[225]
FABP5, S100 A8, RAB2 A, ACTR3, CYB5R1, TGM3, HMGCS2, DEFA1	Colorectal cancer	Tumor exosomes	Plasma	[226]
miR-375, miR-21, miR-210, PD-L1 mRNA	Breast cancer	Tumor exosomes	Blood	[227]
hsa-miR-18a-3p	Breast cancer	Circulating exosomes	Serum	[228]
lncRNA H19	Breast cancer	Serum exosomes	Blood	[229]
miR-21, miR-27a, miR-375	Breast cancer	Serum exosomes	Blood	[230]
miR-424, miR-423, miR-660, let7-i	Breast cancer	Urinary exosomes	Urine	[231]

Compared to conventional biomarker detection techniques, utilizing exosomes as biomarkers for disease diagnosis offers several benefits. Firstly, exosomes can transport multiple biomarkers, enhancing sensitivity [232]. Some of these biomarkers are cell-specific, ensuring high specificity [233]. Secondly, exosome collection can be achieved using standard body fluid methods, eliminating the need for invasive procedures such as tissue sectioning or puncture [234]. Lastly, collected exosomes can be stored long-term through techniques like cryopreservation, enabling subsequent analysis [235].

### Exosomes as therapeutic agent

Exosomes are increasingly studied as therapeutic agents due to their biocompatibility, targeting abilities, and capacity to transport various bioactive molecules, including RNA, proteins, and lipids [236]. Exosomes are proficient at delivering these molecules to target cells, thereby regulating cellular functions. This attribute has demonstrated immense potential in gene therapy, immune regulation, and disease repair [237]. Furthermore, exosomes can be engineered to carry specific therapeutic molecules like RNA therapy, protein drugs, and antibodies, thereby augmenting their therapeutic impact. The structural properties of exosomes provide them with remarkable stability in vivo, and their ability to target specific sites via blood circulation significantly reduces side effects [237]. Additionally, the exploration of exosomes in treating acute inflammation, chronic inflammation, and tumors is an ongoing endeavor. In conclusion, given their wide-ranging application prospects as therapeutic agents, exosomes are anticipated to emerge as a novel type of precise treatment tool (Table 2).

**Table 2:** Exosomes as therapeutic agent in inflammation and cancer

Origin	Biological functions	Disease	Reference
ADSCs	Inhibiting cell proliferation, migration, and inflammatory responses. Inducing immune tolerance	Rheumatoid Arthritis	[238]
DCs	Reducing the toxicity of Triptolide and induce immune suppression	Rheumatoid Arthritis	[239]
Neutrophils	Regulating the inflammatory environment, reduce joint damage in RA, and significantly improve cartilage destruction and overall severity of arthritis	Rheumatoid Arthritis	[240]
iMSCs	Enhancing TGF—β 1 production, induce Th2 and M2 polarization, and reduce the concentration of pro-inflammatory cytokines to alleviate cartilage damage in RA	Rheumatoid Arthritis	[241]
Metabolically engineered stem cell	Targeting reprogramming of macrophages effectively induces anti-inflammatory response in a mouse collagen induced arthritis model	Rheumatoid Arthritis	[242]
Curcumin loaded and R9 peptide modified M2 macrophage	Anti-inflammatory and pro M2 macrophage repolarization	Rheumatoid Arthritis, Spinal Cord Injury	[243]
MSCs	Inhibiting the proliferation and migration of fibroblast like synovial cells, and promoting their apoptosis	Rheumatoid Arthritis	[244]
GMSCs	Significantly inhibit the harmful effects of RASF; Potential to inhibit the invasive migration and spread of RASF	Rheumatoid Arthritis	[245]
MSCs	Inhibiting the activation, migration, and invasion of RA FLSs, and reducing the expression of immune factors	Rheumatoid Arthritis	[246]
hUCMSCs	Inhibiting the proliferation, migration, and invasion of RASFs, improving joint inflammation, and alleviating pathological synovitis	Rheumatoid Arthritis	[247]
BM-MSCs	Inducing macrophages to transition to an anti-inflammatory phenotype, enhancing their ability to engulf apoptotic cell debris, and promoting recruitment of regulatory T cells	Systemic Lupus Erythematosus	[248]
MSCs	Transmitting tsRNA-21109 inhibits M1 polarization of macrophages	Systemic Lupus Erythematosus	[249]
MSCs	Reducing blood glucose levels and increasing plasma insulin levels	Type 1 DM	[250]
UCMSCs	Regulating the abnormal proliferation, apoptosis, and differentiation of CD4 + T cells in peripheral blood of patients with primary Sjogren’s syndrome	Primary Sjogren’s Syndrome	[251]
LGMSCs	Improving saliva secretion function and reducing inflammation	Sjogren’s Syndrome	[252]
OE-MSCs	Regulating the function of MDSCs significantly enhances their immunosuppressive ability, Promoting the secretion of IL-6 and activating the STAT3 pathway	Sjogren’s Syndrome	[253]
MSCs	Promoting the proliferation and function of pro-inflammatory T cells, enhancing the function of regulatory T cells, and alleviating inflammatory reactions	Multiple Sclerosis	[254]
Resveratrol-loaded Macrophage	Targeting central nervous system microglia alleviate multiple sclerosis	Multiple Sclerosis	[255]
UCMSCs	Suppressing peripheral blood mononuclear cell proliferation	Multiple Sclerosis	[256]
hUC-MSCs	Repairing mucosal barrier, restoring intestinal immune homeostasis, and regulating Th2/Th17 cell balance	Inflammatory Bowel Disease	[80]
T cells	Transmitting miR-195a-3p can alleviate the severity of inflammatory bowel disease (IBD) and promote the repair of intestinal epithelial barrier damage	Inflammatory Bowel Disease	[257]
MSCs	Transporting microRNA-181a can alleviate inflammation in DSS-induced colitis mouse models and protect intestinal barrier function	Inflammatory Bowel Disease	[258]
Endotheliocyte	downregulating LPS induced macrophage pro-inflammatory cytokine production and upregulating anti-inflammatory cytokine IL-10	Sepsis	[259]
ADMSCs	Inhibiting the secretion of IL-27 in macrophages to alleviate lung injury caused by sepsis	Sepsis	[74]
EPCs	Reducing pathological damage to kidney tissue, decreasing serum inflammatory response, and decreasing apoptosis and oxidative stress response in kidney tissue	Sepsis	[260]
Docetaxel-loaded M1 macrophage	Activating tumor immune microenvironment to achieve significant inhibitory effect on breast cancer	Breast Cancer	[261]
Engineered exosomes loaded with miR-449a	Inhibiting the proliferation of A549 cells and promoting their apoptosis	Lung Cancer	[262]
M2 Macrophage	Upregulating miR-26a or downregulating AFAP1-AS1 can reverse their effects on EC cell migration and invasion	Esophageal Cancer	[263]
BM-MSCs	Effectively targeting pancreatic cancer, and building a platform for joint delivery of paclitaxel (PTX) and gemcitabine monophosphate (GEMP)	Pancreatic Cancer	[264]
HEK293 Cells	ExomiR-34a synthesized by ultrasonic method can significantly inhibit the growth of pancreatic cancer in vivo and in vitro	Pancreatic Cancer	[265]
hCD-MSCs	Expressing TRAIL and enhancing the specific killing effect on cancer cells	Cervical Carcinoma	[266]
TAMs	Carrying miR-660-5p inhibit ferroptosis in cervical cancer cells	Cervical Carcinoma	[267]

### Exosomes as threapeutic carries

Exosomes hold promise as natural platforms for drug loading and delivery, complementing their inherent effects. Exosomes exhibit structural stability, and desired cargos can be loaded through physical, chemical, or biological approaches. The lipid bilayer safeguards internal biomolecules or drugs from enzymatic degradation, including RNase, thereby minimizing their loss [268]. Exosomes can be delivered via intranasal, intravenous, intraperitoneal, and intracranial routes. They can traverse the blood–brain barrier, enabling drug delivery to the brain [269]. Exosomes provide a non-toxic alternative. Exosomes demonstrate lower immunogenicity and enhanced pharmacokinetic and pharmacodynamic characteristics relative to synthetic nanocarriers [270]. The advantages of exosomes have made them a focal point in drug delivery system research (Table 3).

**Table 3:** Exosomes as therapeutic carries used for diagnosis and treatment monitoring in inflammation and cancer

Disease	Exosomes type	Reference
Sepsis	Engineered exosomes, HUCCMS exosomes	[271–273]
Colitis and Rheumatoid arthritis	Dendritic cell exosomes	[239]
Rheumatoid arthritis	Serum exosomes Engineered exosomes	[242, 243, 274–276]
Systemic lupus erythematosus	Engineered exosomes, MSC exosomes	[277, 278]
Diabetes mellitus	Engineered exosomes	[279]
Sjogren’s syndrome	Engineered exosomes	[280, 281]
Multiple sclerosis	Macrophage exosomes、MSC exosomes	[256, 282]
Spinal cord injury	M2 exosomes	[283]
Kidney Diseases	Engineered exosomes	[284]
Periodontitis	Engineered exosomes	[285, 286]
Osteoarthritis	OA-FLS exosomes	[287]
Inflammatory bowel disease and Colitis-Associated cancer	Engineered exosomes, Plant-Derived exosomes	[288, 289]
Breast cancer	Tumor exosomes	[290–292]
Pancreatic cancer	Tumor exosomes, Engineered exosomes	[224, 293–296]
Lung diseases	MSC exosomes, Engineered exosomes, CAR-T exosomes	[297–300]
Colorectal cancer	Engineered exosomes, Tumor exosomes	[301–307]
Liver cancer	Engineered exosomes	[308, 309]
Gastric cancer	Tumor exosomes, si–c-Met exosomes	[310, 311]
Esophageal squamous cell carcinoma	UCMSCs exosomes	[312]

### Clinical trails

Exosomes hold significant roles for inflammation and cancer. Recent data obtained from www.clinicaltrials.gov and www.chictr.org.cn, which are outside the pre-clinical stage, provide detailed information on the timeline, type, effect, and study population of secreted research data (Table 4).

**Table 4:** Clinical trials of inflammation and cancer

ID	Title	Exosome Types	Population	Disease	Effect	Status
ChiCTR2100048082	Identification of Serum and Exosome Markers in Lupus Nephritis	Serum exosomes	SLE patients	Systemic lupus erythematosus	Diagnosis	Prospective registration
NCT04894695	Urine Exosomes to Identify Biomarkers for LN	Urine exosomes	Healthy controluik; systemic lupus erythematosus patients; lupus nephritis patients	Lupus nephritis	Diagnosis	Unknown status
ChiCTR2400082826	Expression of plasma exosome protein as a potential biomarker and pathway study in patients with sepsis-related encephalopathy	Plasma exosome	Patients diagnosed within 48 h of sepsis; 18–80 years old	Sepsis-associated encephalopathy	Diagnosis	Prospective registration
ChiCTR2200058506	The value of plasma exosome level in early diagnosis and prognosis of septic patients	Plasma exosome	Patients aged > 18 years, admitted to ICU with sepsis	Sepsis	Diagnosis	Prospective registration
ChiCTR2100050553	Serum exosome proteomics in patients with sepsis: a prospective single-center cohort study	Serum exosome	Sepsis patients	Sepsis	Diagnosis	Prospective registration
NCT04979767	Function of Circulating Exosomes in Sepsis-induced Immunosuppression	Circulating Exosomes	≥ 50 years with ≥ 2 chronic comorbidities	Sepsis	Diagnosis	Unknown status
	Plasma exosome and sepsis induced acute lung injury	Exosomes-ANPEP	Sepsis patients	Acute lung injury in sepsis	Therapy	Prospective registration
ChiCTR2400080810	Application of multi-omics analysis of exosomes in early diagnosis and risk stratification of ARDS in sepsis	Exosomes in the Balf	Age ≥ 18 years; Sepsis patients	Sepsis-associated acute respiratory failure syndrome	Diagnosis	Prospective registration
ChiCTR2300069120	Clinical study of erythrocyte exosomes in the repair of periodontal and peri-implant tissue defects	Erythrocyte exosomes	Ealthy periodontal tissue or mild chronic periodontitis	Periodontitis/Dental defect	Therapy	Prospective registration
NCT04270006	Evaluation of Adipose Derived Stem Cells Exo.in Treatment of Periodontitis (exosomes)	Adipose derived stem cells exosomes	Advanced periodontitis (stage III or IV); healthy patients	Periodontitis	Therapy	Unknown status
NCT06764004	The Effect of Human Umbilical Cord Mesenchymal Stem Cells and Exosomes on the Healing of Postoperative Pain and Periapical Lesions in the Treatment of Apical Periodontitis: Randomized Controlled Clinical Study	Umbilical cord MSCs exosomes	Patients between the ages of 9–15;	Apical periodontitis	Therapy	Not yet recruiting
NCT06755021	Whey Protein Milk-Derived Exosomes	Milk-Derived Exosomes	Age 18–65 years	Ulcerative colitis	Therapy	Not yet recruiting
NCT04879810	Plant Exosomes ± Curcumin to Abrogate Symptoms of Inflammatory Bowel Disease	Curcumin Exosomes	Patients must have a confirmed diagnosis of IBD	Inflammatory Bowel Disease (IBD)	Therapy	Completed
NCT05499156	Safety of Injection of Placental Mesenchymal Stem Cell Derived Exosomes for Treatment of Resistant Perianal Fistula in Crohn's Patients	Placental MSC exosomes	Age between 18–70 years old; Occurrence of complex perianal fistula	Perianal fistula in patients with crohn’s disease	Therapy	Unknown status
ChiCTR1900020505	Differential analysis for macromolecular noncoding RNAs expression profiles of secretory in peripheral blood of breast cancer	Macromolecular noncoding RNAs	Women aged 18–70 years	Breast cancer	Diagnosis	Retrospective registration
ChiCTR1900026195	Exosomes as potential prognostic and predictive biomarkers to assess the tumor response to neoadjuvant chemotherapy in breast cancer patients	Exosome RNR-Seq	Women aged more than 18 years	Breast cancer	Diagnosis	Prospective registration
NCT01344109	A Pilot Study of Tumor-Derived Exosomes as Diagnostic and Prognostic Markers in Breast Cancer Patients Receiving Neoadjuvant Chemotherapy	Tumor exosomes	Women with biopsy proven invasive carcinoma of the breast	Breast cancer	Diagnosis	Withdrawn
NCT05286684	Feasibility of Exosome Analysis in Cerebrospinal Fluid During the Diagnostic Workup of Metastatic Meningitis (Exo-LCR) (Exo-LCR)	Exo-LCR	Age ≥ 18	Breast Cancer	Diagnosis	Completed
NCT05955521	Exosome as the Prognostic and Predictive Biomarker in EBC Patients	Exosome and ctDNA	Early breast cancer; planned neoadjuvant chemotherapy; triple negative breast cancer or HER2-positive breast cancer	Early breast cancer	Diagnosis	Active, not recruiting
NCT06342414	An Exosome-Based Liquid Biopsy for the Differential Diagnosis of Primary Liver Cancer (ELUCIDAT)	Exo-miRNA	Hepatocellular carcinoma; intrahepatic cholangiocarcinoma	Liver cancer	Diagnosis	Recruiting
ChiCTR2200060611	Detection of exosomes based on flow cytometry	Plasma exsomes	Lung cancer, liver cancer, and breast cancer patients	Lung cancer, liver cancer, breast cancer	Diagnosis	Prospective registration
NCT04529915	Multicenter Clinical Research for Early Diagnosis of Lung Cancer Using Blood Plasma Derived Exosome	Plasma exosomes	40 Years and older (adult, older adult);	Lung cancer	Diagnosis	Unknown status
NCT05854030	Serum Exosomal miRNA Predicting the Therapeutic Efficiency in Lung Squamous Carcinoma	Serum exosomes	18 Years and older (adult, older adult);	Lung squamous carcinoma	Therapy	Recruiting
NCT03542253	Combined Diagnosis of CT and Exosome in Early Lung Cancer	Blood exosomes	At least 18 years of age, male or female;	Early Lung Cancer	Diagnosis	Unknown status
NCT03830619	Serum Exosomal Long Noncoding RNAs as Potential Biomarkers for Lung Cancer Diagnosis	Serum exosomes	Lung cancer patients are 18 to 75 years old,	Lung cancer	Diagnosis	Completed
ChiCTR2100054275	Clinical application of exosomal DNA as a “liquid biopsy” marker for whole process monitoring of liver cancer	Exosome DNA	Hepatocellular carcinoma patients	Hepatic cancer	Diagnosis	Prospective registration
ChiCTR2100051940	A prospective study of exosomal LncSENP6 for early warning of liver metastases in colorectal cancer	Exosome LncSENP6	Colorectal cancer patients	Colorectal cancer	Diagnosis	Prospective registration
ChiCTR2200061592	A multi-center study: the application value of plasma exosomal non-coding RNA in the early screening and clinical progress of colorectal cancer	Plasma exosome	Age 20–80 years old;	Colorectal adenomas and colorectal cancer	Diagnosis	Prospective registration
NCT01294072	Study Investigating the Ability of Plant Exosomes to Deliver Curcumin to Normal and Colon Cancer Tissue	Plant exosomes	20 years and older (adult, older adult);	Colon cancer	Carrier	Recruiting
ChiCTR1800018038	A clinical research about the serum exosome-derived circRNAs function as molecular phynotype biomarker for the boardline resectable pancreatic cancer	Serum exosome circRNAs	Pancreatic cancer patients	Pancreatic cancer	Diagnosis	Prospective registration
ChiCTR1800018331	The diagnostic value of circulating tumor cells and exosomes acquisited from portal venous by endoscopic ultrasound in pancreatic cancer: a prospective clinical study	CTCs exosome	Aged from 18 to 80 years-old;	Pancreatic cancer	Diagnosis	Prospective registration
NCT06108531	Tumor Exosome Liquid Biopsy Strategy to Diagnose Pancreatic Cancer	Peripheral blood exosomes	Pancreatic patients	Pancreatic cancer	Diagnosis	Enrolling by invitation
NCT04636788	Circulating Extracellular Exosomal Small RNA as Potential Biomarker for Human Pancreatic Cancer	Circulating exosome small RNA	Age > 18; pancreatic cancer patients	Pancreatic cancer	Diagnosis	Unknown status
NCT03032913	Diagnostic Accuracy of Circulating Tumor Cells (CTCs) and Onco-exosome Quantification in the Diagnosis of Pancreatic Cancer—PANC-CTC (PANC-CTC)	Onco-exosome	Suspicion or recent diagnosis of PDAC	Pancreatic cancer	Diagnosis	Completed
ChiCTR2300072187	Identification of exosomal circular RNA circSLC26 A4 as a liquid biopsy marker for cervical cancer	Exosome circSLC26 A4	Cervical squamous cell carcinoma	Cervical cancer	Diagnosis	Prospective registration
NCT06278064	Exosome-based Liquid Biopsies for Upper Gastrointestinal Cancers Diagnosis	Plasma exosomes	Upper gastrointestinal cancers or benign upper gastrointestinal diseases	Esophagus cancer; gastric cancer	Diagnosis	Recruiting
NCT03488134	Predicting Prognosis and Recurrence of Thyroid Cancer Via New Biomarkers, Urinary Exosomal Thyroglobulin and Galectin-3	Urinary exosomes	20 Years to 80 Years (Adult, Older Adult);	Thyroid cancer	Diagnosis	Completed
NCT05463107	Correlation Between Various Urinary Exosomal Protein Biomarkers and Pathological Manifestation in Thyroid Follicular Neoplasm: Early and Pre-operative Diagnosis of Follicular Thyroid Cancer	Urinary exosomes	20 Years to 80 Years (Adult, Older Adult);	Thyroid cancer	Diagnosis	Recruiting
NCT06469892	Saliva and Plasma Exosomes for Oral Leukoplakia Malignant Transformation Diagnosis and Oral Cancer Prognosis Monitoring	Saliva and plasma exosomes	30 Years to 80 Years (Adult, Older Adult); Patients with Oral Cancer	Oral cancer	Diagnosis	Completed

Data sources – clinical registration website and the Chinese clinical trial registry website

## Challenge and perspectives

### Technical and methodological challenges

Recent advancements in exosome separation technology have greatly enhanced the understanding of their components. However, the inherent complexity of biological samples, coupled with their overlapping physicochemical and biochemical characteristics, renders exosomes themselves notably complex [313]. Moreover, exosomes intrinsically exhibit heterogeneity. These factors collectively present considerable challenges to the rapid and effective separation of exosomes [314]. The common isolation methods for exosomes include ultracentrifugation, density gradient centrifugation, immunoaffinity, ultrafiltration, and microfluidic technology. Among them, ultracentrifugation is the most widely used method [315]. However, each of these methods has its own drawbacks. For instance, ultracentrifugation has a low purity and is prone to contamination with other cellular secretions. It is also cumbersome and time-consuming, making it unsuitable for high-throughput screening. Moreover, the yield of exosomes obtained through this method is relatively low, which may not meet clinical demands [316, 317]. Considering the limitations of the method, its inability to effectively separate different subpopulations of exosomes may affect the accuracy of downstream applications. In order to overcome the shortcomings of exosome separation methods, the combination of new technologies and separation methods has become a trend, aiming to find a method with low separation cost, high purity, fast speed, high throughput, and high recovery rate [316].

Exosomes originate from a multitude of sources, conferring upon them considerable heterogeneity. Even exosomes secreted by identical cell types can exhibit diverse biological characteristics due to variations in secretion conditions, such as stress or disease states [318]. Furthermore, considering the differences in size, morphology, and molecular composition of exosomes, the identification of exosomes is relatively complex. Exosome identification methods encompass transmission electron microscopy (TEM), scanning electron microscopy (SEM), cryo-electron microscopy, and atomic force microscopy (AFM), each offering distinct benefits and limitations [319]. SEM is presently the preeminent method for exosome identification, providing crucial insights into their morphology and structure. This technique also yields compositional data and diverse crystal defect information. Furthermore, SEM exhibits remarkable resolution, enabling the direct observation of exosomes’ varying sizes and forms [320]. The subsequent identification of exosomes components necessitates the utilization of Western Blotting, flow cytometry, or immunoelectron microscopy. These techniques are essential for characterizing the protein constituents of exosomes [321]. However, a universally accepted standard for the identification of exosomes remains elusive. While certain markers, such as CD9, CD63, and CD81, are commonly used to identify exosomes protein components, their specificity and sensitivity remain subjects of debate [322]. Future endeavors should prioritize the establishment of a standardized procedure for exosome identification, encompassing the selection of exosomes markers, the standardization of extraction techniques, and the harmonization of detection platforms [323].

Exosome analysis employs quantitative techniques including nanoparticle tracking analysis, flow cytometry, tunable resistive pulse sensing, electron microscopy, dynamic light scattering, and the use of microfluidic devices [324]. Among these, Nanoparticle Tracking Analysis (NTA) stands out as the most frequently employed. NTA operates based on the principle of light scattering, enabling the determination of size distribution and concentration of nanoparticles in suspension through the measurement of their displacement due to Brownian motion. Noted for its simplicity, speed, and heightened sensitivity, NTA offers precise data on particle size and concentration, making it especially suitable for the quantitative analysis of exosomes. The diminutive size of exosomes presents significant challenges to conventional quantitative methodologies, which tend to be both cumbersome and imprecise [325]. Furthermore, obtaining pure exosome samples for separation is fraught with difficulties, and the limited efficiency of current technologies often results in co-precipitation with non-exosome molecules and vesicle structure damage [326]. Most pressingly, the requisite instruments are costly, demand rigorous maintenance, and pose practical adoption challenges in resource-constrained environments. However, as technology evolves, emerging methods like resistive pulse sensing and tuned resistive pulse sensing are expected to gain traction in the quantitative analysis of exosomes. These novel techniques promise enhanced sensitivity coupled with minimal sample requirements, thereby bolstering the potential of exosomes in clinical diagnostics and biomedical research [327, 328].

### Barriers to clinical translation

Exosomes, pivotal biomolecular carriers, exhibit immense potential for clinical applications. Nonetheless, challenges persist in their translation to clinical use, particularly in regulatory matters, production standardization, and quality assurance [329]. Exosomes, which contain a variety of functional biomolecules, significantly impact the body in numerous ways that are yet to be fully understood. To enhance our comprehension of exosomes and their mechanisms, it is imperative to closely examine their specific components and potential processes. This will aid in determining their individual biosafety characteristics and quality control measures. The mass production of exosomes for medical use holds significant potential. The large-scale production of GMP-grade exosomes is inherently complex. Even when using the same cell source, the final composition of exosomes can differ due to variations in growth conditions such as temperature, medium composition, and supplementation [330]. This poses significant challenges for the mass production of exosomes. Additionally, the process of manufacturing exosomes on a large scale, including subsequent filling steps, as well as monitoring storage conditions and controlling distribution channels, must all be conducted in compliance with regulatory and GMP standards. As previously noted, the processes of exosome separation, identification, and quantification lack a unified standard. Each methodology presents its unique strengths and weaknesses, leading to significant result variations across different laboratories. The challenge lies in selecting the most suitable separation method and ensuring consistent quality during large-scale production for clinical translation of exosomes. At present, exosome production predominantly depends on cell culture. While cell culture is inherently viable, transitioning it from a laboratory scale to mass production encounters hurdles in various aspects, including cell culture systems, culture mediums, and production environments [331].

### Future research directions

Recent research has demonstrated the pivotal role of exosomes and their cargo in a range of clinical diseases, with current focus primarily on diagnosis, treatment, and drug delivery. The future of exosome diagnosis lies in the potential changes in the biological components contained within exosomes under varying disease states. These changes could provide unique biomarker information that could reflect disease status more accurately and promptly. Therefore, the discovery of new biomarkers is one of the main directions for the development of future exosome research. In the future personalized treatment of exosomes, exosomes have individual characteristics that can reflect the specific disease status or treatment response of patients. Future research will explore the application of exosomes in personalized treatment, including customizing treatment plans based on exosome components, monitoring treatment effects, and evaluating patient responses to treatment. Regarding the future of exosome-mediated drug delivery, the natural biocompatibility and targeting capability of exosomes render them perfect candidates for drug carriers. Potential areas for further research may include optimizing the mechanisms by which drugs are loaded and released by exosomes, as well as utilizing exosomes for gene therapy, RNA interference, and the delivery of antibody drugs.

## Conclusion

In summary, exosomes, as key mediators of intercellular communication, provide a whole new direction to elucidate the mechanism of inflammation and cancer. Specifically, in inflammatory diseases, exosomes regulate immune responses by modulating macrophage polarization, cytokine secretion, and microenvironment interactions, playing either pro- or anti-inflammatory roles depending on their cargo; in cancer, exosomes coordinate TME remodeling and promote angiogenesis, metastasis, and immune evasion via interacting with ECM, CAF, immune cells, and blood vessels, while also serving as tumor-specific biomarkers for noninvasive liquid biopsy. Exosomes with their intrinsic biocompatibility, low immunogenicity, and carrying various bioactive molecules make them versatile tools for diagnostics, therapeutics, and drug delivery. Clinically, exosomes show great promise in early disease detection, prognostic therapy, and drug delivery, and ongoing clinical trials validate their efficacy in multiple inflammatory disorders and cancers.

However, there remain challenges in standardizing isolation, quantification, and characterization protocols to address exosome heterogeneity. Scale production under GMP guidance, long-term biosafety evaluations, and ethical considerations regarding cell source remain critical barriers to clinical translation. Future research should prioritize elucidating exosome-mediated signaling networks, optimizing cargo loading strategies, and exploring personalized exosome-based therapies to fully realize their potential in precision medicine. Resolving these challenges will pave the way for exosomes to revolutionize the diagnosis and treatment of inflammation and tumors.

## Data Availability

Not applicable.
